# Alcohol, Aggression, and Violence: From Public Health to Neuroscience

**DOI:** 10.3389/fpsyg.2021.699726

**Published:** 2021-12-20

**Authors:** Kajol V. Sontate, Mohammad Rahim Kamaluddin, Isa Naina Mohamed, Rashidi Mohamed Pakri Mohamed, Mohd. Farooq Shaikh, Haziq Kamal, Jaya Kumar

**Affiliations:** ^1^National Forensic Sciences University, Gandhinagar, India; ^2^Centre for Research in Psychology and Human Well-Being, Faculty of Social Sciences and Humanities, Universiti Kebangsaan Malaysia, Bangi, Malaysia; ^3^Department of Pharmacology, Faculty of Medicine, Universiti Kebangsaan Malaysia Medical Centre, Kuala Lumpur, Malaysia; ^4^Department of Family Medicine, Faculty of Medicine, Universiti Kebangsaan Malaysia Medical Centre, Kuala Lumpur, Malaysia; ^5^Neuropharmacology Research Laboratory, Jeffrey Cheah School of Medicine and Health Sciences, Monash University Malaysia, Subang Jaya, Malaysia; ^6^Department of Physiology, Faculty of Medicine, Universiti Kebangsaan Malaysia Medical Centre, Kuala Lumpur, Malaysia

**Keywords:** alcohol, aggression, public health, brain, domestic, violence, violent, serotonin

## Abstract

Alcohol has been associated with violent crimes and domestic violence across many nations. Various etiological factors were linked to chronic alcohol use and violence including psychiatric comorbidities of perpetrators such as personality disorders, mood disorders, and intermittent explosive disorders. Aggression is the precursor of violence and individuals prone to aggressive behaviors are more likely to commit impulsive violent crimes, especially under the influence of alcohol. Findings from brain studies indicate long-term alcohol consumption induced morphological changes in brain regions involved in self-control, decision-making, and emotional processing. In line with this, the inherent dopaminergic and serotonergic anomalies seen in aggressive individuals increase their susceptibility to commit violent crimes when alcohol present in their system. In relation to this, this article intends to investigate the influence of alcohol on aggression with sociopsychological and neuroscientific perspectives by looking into comorbidity of personality or mood disorders, state of the mind during alcohol consumption, types of beverages, environmental trigger, neurochemical changes, and gender differences that influence individual responses to alcohol intake and susceptibility to intoxicated aggression.

## Introduction

Alcohol use disorder (AUD) is one of the leading causes of the global burden of disease and injury (WHO), despite the continuous discovery of novel pharmacotherapeutic agents (Pakri Mohamed et al., 2018). Various factors such as environmental, social, situational, and cultural context have distinctive consequences toward substance use and its effects on individuals (Latkin et al., 2017). Alcohol alters the mental state of individuals, including emotional processing and rational thinking, making the users unpredictable and dangerous, especially young people (Australian Government, 2017) or those with pre-existing psychological or psychiatric comorbidities (Brem et al., 2018; Puhalla et al., 2020). Violence related to substance use has been widely reported and studied, particularly the potential for violent outcomes between the different substances of use (Duke et al., 2018). Studies from various countries have reported crimes and domestic violence owing to alcohol (Hagelstam and Häkkänen, 2006; Mayshak et al., 2020), especially during the recent state of global coronavirus disease 2019 (COVID-19) pandemic (Finlay and Gilmore, 2020).

## Alcohol and Domestic Abuse/Violence

There is a strong evidence linking alcohol with domestic abuse or domestic violence (Gadd et al., 2019). A study conducted within the metropolitan area of Melbourne, Australia found that alcohol outlet density was significantly associated with domestic violence rates over time (Livingston, 2011). In Australia, alcohol-related domestic violence is twice more likely to involve physical violence including life-threatening injuries (Mayshak et al., 2020). In the UK police report audit, approximately two-thirds of domestic incidents reported to police involve “under the influence of alcohol” (Alcohol Research UK). The same study also noted more aggression if alcohol was involved and persons involved considered alcohol to have a direct effect on their behavior. International evidence reveals a similar pattern with men tend to cause worse assaults after drinking and women are more likely to suffer from abuse with living partners who are heavy drinkers (Reno et al., 2010; Graham et al., 2011). These behavioral patterns cannot be inferred from women. Studies have demonstrated that women who are heavy alcohol drinkers tend to suffer from abuse themselves and also suffer from higher aggression from their partners (Hutchison, 1999; Iverson et al., 2013). In India, those who had a heavy drinker in their lives (family, relative, neighbor, etc.) reported having been harmed by them through physical, sexual, psychological, financial, and social. In Kerala, India, a cross-sectional study involving spouses of alcohol-dependent males undergoing a deaddiction program reported a high correlation between domestic violence and years of marriage and the number of stressful events in the past year (Indu et al., 2018). In the USA, 40% of the reported domestic violence has the alcohol factor present during the time of the offense (Galbicsek, 2020). It is also found that the intensity of violence is greater when the offender is intoxicated compared to when he/she is not. Based on existing literature, alcohol consumption is more related to the severity of domestic violence rather than its occurrence (Graham et al., 2011) and exacerbated by an increase in consumption (Ferrari et al., 2016). Although there is a clear correlation between alcohol and domestic abuse, these correlations are limited to men and, therefore, form a complex relationship, hence establishing a unidirectional relationship between domestic violence and alcohol is not possible at present (De Paula Gebara et al., 2015). As per UN Women of the United Nations, the global prevalence of domestic violence against women was 1 in 3 prior to COVID-19 pandemic, mainly perpetrated by their intimate partners. Emerging data from a number of countries show an increase in calls to domestic violence helplines since the beginning of COVID-19 pandemic. The United Nations Secretary-General has referred to this surge in domestic violence amid COVID-19 pandemic as a “shadow pandemic” (Women UN, 2020). Several countries showed a shockingly increasing pattern of domestic violence cases globally, up to 50% in Brazil, 20% rise in helpline calls in Spain, 30% in Cyprus (The Guardian, 2020), 25% increase in helpline calls and about 150% rise in Refuge website in the UK (Bradbury-Jones and Isham, 2020), and almost doubled cases of domestic violence in the Hubei, China (Anju, 2019). COVID-19 pandemic-induced increase in global domestic violence was irrespective of the economic status of the countries (Finlay and Gilmore, 2020). In line with this, the alcohol sales in March 2020 were increased by 67% in the UK during lockdown (Finlay and Gilmore, 2020). Contrary to this, a recent systematic review revealed that there is insufficient evidence to suggest that COVID-19 pandemic has led to increased substance use and domestic violence (Abdo et al., 2020) (Table 1).

**Table 1:** Alcohol and domestic violence.

**Study**	**Objective**	**Method**	**Sample size**	**Findings**	**Country (where the data is from)**
Kwagala et al. (2013)	The impact of women's empowerment and partners' behaviors on IPPV among married women in Uganda was investigated in this study.	Data from Uganda's 2011 Demographic and Health Survey were used. Cross tabulations (chi-square tests) and multivariate logistic regressions were employed.	1,307 women	Male partner habits, dominant behavior associated with perceived infidelity, and getting intoxicated are the factors that are specifically linked to Intimate Partner Physical Violence in Uganda.	Uganda
Kazzaz et al. (2019)	To look at the prevalence, risk factors, and effects of domestic violence in Saudi Arabia.	Systematic review utilizing PRISMA guidelines	–	Domestic abuse affects at least one out of every three women (DV). Low levels of education and alcohol/drug abuse appeared as a major risk factors associated with DV.	Saudi Arabia
Guclu and Can (2018)	In order to determine the frequency and risk factors of domestic violence in a multi-cultural region of Turkey, researchers conducted a study.	Cross-sectional study	602 women	Domestic violence has been shown to be highly influenced by alcohol use and race.	Turkey
Adjah and Agbemafle (2016)	To find characteristics that enhanced the likelihood of a domestic abuse incident as reported by Ghanaian women who had never married.	A multivariate logistic model was used to analyze data from the 2008 Ghana Demographic and Health Survey (GDHS), and risk factors were identified using the forward selection technique.	1,524 ever married women	A woman is found to be at a greater risk of domestic abuse in the following conditions—at her home, her husband's alcohol consumption, and her family history of violence. Women whose husbands drink alcohol are 2.5 times more likely to experience sexual violence than women whose husbands do not drink alcohol.	Ghana
Curtis et al. (2019)	To evaluate major differences between three types of violence: family violence, intimate partner violence (IPV), and other violence, as well as the association between alcohol use and FDV in the Australian community.	A stratified random sample strategy was used to conduct an online panel survey.	5,118 participants	In several cases of Family and Domestic Violence, especially Intimate Partner Violence, alcohol plays a prominent role. At IPV cases, drinking alcohol was linked to an increased risk of physical assault and injuries.	Australia
Maffli and Zumbrunn (2003)	To look at domestic violence incidents that have been reported to the police, with an emphasis on the drinking habits of those involved.	Interviews containing A standardized questionnaire were conducted with the police and victims	42 cases	Officers discovered that one or more of the people involved in the domestic violence act were intoxicated by alcohol in 40% of the 42 cases they examined (a minimum of 33% of the offenders and 10% of the victims).	Switzerland
Devries et al. (2014)	The purpose of this study was to look at the evidence of a link between IPV victimization and female alcohol use.	A systematic review and meta-analysis of Cross-sectional and longitudinal studies.	–	Evidently, a strong connection is seen between alcohol consumption and female victims of intimate partner physical or sexual assault.	Multiple – countries.
Semahegn et al. (2013)	To find out how often domestic violence is among married women of reproductive age in northwestern Ethiopia, as well as what factors predict it.	Community based cross-sectional study.	682 married women and 46 key informants	Domestic abuse was predominant, and the husband's alcohol intake, decision-making authority, annual household income, and becoming pregnant were all determinants or predicting factors.	Ethiopia
Ali et al. (2014)	To look at the prevalence of current (within the last year) domestic abuse and the socio-demographic variables that are linked to domestic violence against women.	Cross sectional household survey (in person interview). Multivariable analysis was done.	1,009 Women	Domestic violence was observed to be far more widespread in eastern Sudan, with the husband's alcohol intake being one of the strongest links to educational status and polygamy.	Sudan
Leite et al. (2019)	To investigate whether there's a link between intimate partner's socio-demographic and behavioral traits and their	Cross-sectional study. The Pearson c2 test was used for bivariate analysis, while Poisson regression with robust variance	938 women (20–59 years) in intimate relationship	Controlling men who drank alcoholic beverages were found to be more likely to commit psychological and physical abuse.	Brazil
	history of violence toward women.	was used for multivariate analysis.			
Begum et al. (2015)	The incidence and determinants of domestic violence among women in Mumbai's urban slums were investigated in this study.	A community-based cross-sectional household survey of eligible women was conducted.	1,137 married women (18–39 years of age)	Domestic violence was prevalent in urban slums and husbands use of alcohol, early marriage, working status, justified wife beating were the factors significantly associated with domestic violence.	India
Indu et al. (2018)	To determine the prevalence of domestic violence in the wives of alcohol-dependent males who attended a tertiary care hospital in South Kerala's de-addiction center.	Cross-sectional study	60 Participants	The wives of alcoholic men were observed to be experiencing domestic violence and psychiatric morbidity at a higher rate.	India
Pewa et al. (2015)	The purpose of this study was to assess the prevalence of domestic violence and the influence it has on dental health.	Observational cross-sectional study	150 married women (18–60 years)	Alcoholism was the most important determinant for domestic abuse, complemented by the level of literacy and having a girl child.	India
Kaufmann et al. (2014)	To investigate if alcohol intake was considerably higher in the clinical sample of substance-abusing women prior to violent vs. peaceful relationship conflict episodes.	At the start of the trial, as well as at the 6-, 12-, and 18-month follow-ups, study participants gave data on drug use, individual and relationship functioning *via* interviews and questionnaires.	277 women (18–49 years)	For this clinical study of substance-abusing women, alcohol intake was substantially higher prior to aggressive than peaceful partnership dispute cases.	America
Livingston (2011)	Examining the domestic violence at the population level, with a specific focus on the longitudinal link between alcohol availability and domestic violence rates at the neighborhood level.	Cross-sectional time-series	Data was collected for 186 post-codes in Melbourne's metropolitan region (1996–2005)	Over time, the density of alcohol outlets was found to be strongly linked to rates of domestic abuse.	Australia
Smith et al. (2012)	This research looked into Intimate Partner Violence perpetration and victimization in the context of alcohol, cannabis, cocaine, and opioid use disorders.	The National Epidemiologic Survey on Alcohol and Related Conditions, wave two (2004–2005), was used to evaluate the data. Logistic regression models were used to examine associations between drug use disorders and IPV while adjusting for significant variables and taking into consideration the survey's complicated design.	43,093 (civilian, non-institutionalized adult population in the United States)	Intimate partner abuse was frequently related to drug use disorders, with alcohol and cocaine use disorders being the most closely correlated with IPV offense.	America
Lewis et al. (2015)	The connection between emotional distress (depression, brooding, and negative affect), alcohol effects, and bidirectional intimate partner violence among lesbian women was investigated in this study.	Negative affect, Rumination, Depressive symptoms were measured.	414 lesbian women (18–35 years)	The self-medication theory states that lesbian women who are more emotionally distressed are more likely to indulge in drinking to cope and thus, exhibit alcohol abuse and related issues. These alcohol-related consequences were linked to bidirectional partner abuse.	America
Renzetti et al. (2018)	To look at how ambivalent sexism (hostile and benevolent sexism) affect the link between alcohol consumption and IPV perpetration.	Survey method was used and several scales were administered (demographic, ambivalent sexism scale, Collaborative Studies on Genetics and Alcoholism (COGA) Study, Severity of Violence Against Women Scale)	255 community-based men	Higher levels of alcohol intake and hostile sexism are both attributed to IPV perpetration, with sexual violence moderating the alcohol–IPV relationship for physical IPV but not for psychological IPV. Furthermore, for men with low levels of hostile sexism, excessive amount of alcohol intake has a greater effect on physical IPV perpetration than for men with high levels of hostile sexism.	America

## Alcohol, Aggression, and Violence: Psychiatric Comorbidities

There were various publications related to the etiological factors associating alcohol use and violence. Study has shown that alcohol was most commonly abused among adolescents and school children (Bland et al., 2018). Factors such as developmental milestones when a child is growing up can predict violence and substance abuse in adults (Hentges et al., 2018; Malti, 2020). Retrospectively, heavy drinking in later life can be predicted by early childhood aggression (Gottfried and Christopher, 2017). A combination of substance use and psychiatric disorders is associated with an above-average risk of adult violent behavior (Wiener et al., 2018). Mental disorders such as anxiety and mood disorders have also been commonly associated with AUD (Gimeno et al., 2017). AUD and depressive symptoms are commonly reported with other mood disorders and have greater severity and worse prognosis compared when it is concomitant with AUD (Higley and Linnoila, 2002). There are possibilities of the symptoms exhibited by the patients during withdrawal or acute intoxication that are pre-existing effective disorders or in a combination (Serafini et al., 2017). The most common symptoms of substance withdrawal include agitation. Other symptoms such as disinhibition and despair are commonly associated with substance abuse disorder that would be amplified into self-destructive acts and impulsivity (Goldstein et al., 2017; Duica et al., 2020). In addition, men with antisocial traits are at greater risk of binge alcohol consumption and commit intimate partner violence (Brem et al., 2018). In US, the prevalence of antisocial personality disorder and adulthood antisocial behavioral syndrome was 4.3 and 20%, respectively, and both the syndromes were significantly associated with 12-month and lifetime substance use (Goldstein et al., 2017). Based on a study conducted in Italy that had a population of 717 make subjects−404 alcoholics and 282 having a personality disorder, alcohol consumption was higher among those who suffer from psychiatric conditions, especially personality disorder (39%; antisocial personality disorder at the most) and 14.2% have a dual diagnosis (personality disorder and alcohol dependence). The antisocial personality population (more than any other personality disorder) had an early onset of alcohol abuse and its association with physical dependence and legal problems (Poldrugo, 1998). Similar studies were conducted in the prisons of North Italy, which also suggested that there is a positive correlation between AUD and personality disorders and the risk of engaging in criminal acts is higher within the individuals with dual diagnosis (alcoholics and sociopaths). Intermittent explosive disorder (IED), characterized by repeated, sudden explosive outbursts of anger or violence, has been associated with a history of childhood abuse and AUD is at a greater risk for intoxicated aggression (Puhalla et al., 2020) and also to develop substance use disorder compared to those without IED (Coccaro et al., 2017).

## Alcohol, Aggression, and Crime

Aggression is the basic ingredient of acts of violence (Eisner and Malti, 2015). Violence as aggression has the goal of extreme harm including death. In this context, violent and criminal behavior is often associated with substance abuse (Anderson and Bushman, 2002). Alcohol is one of the major ingredients of violent incidents (i.e., murder) due to its disinhibiting effects along with loss of emotional control that increases the susceptibility to physical assaults and eventually murder (Karlsson, 1998). According to Mokdad et al. (2004) and Pinel and Barnes (2018), alcohol is involved in more than 2 million deaths (deaths due to ill health, accidents, and violence) each year across the world. A moderate dose of alcohol in the blood tends to cause cognitive, perceptual, verbal, and motor impairments as well as a loss of control, which eventually lead to unacceptable social behavior including violence (Pinel and Barnes, 2018). From a criminological perspective, alcohol is an important factor in violent interactions that culminate in murder (Wahlsten et al., 2007). Substance abuse, especially alcohol, is widely acknowledged as an important risk marker for criminal behavior and violent crimes including those with mental disorders (Brennan et al., 2000; Wallace et al., 2004; Erkiran et al., 2006). The strong link between alcohol use and violence is well-demonstrated (Mann et al., 2006), as alcohol consumption is an important factor for the prevalence of violence (Room and Rossow, 2001).

Alcohol facilitates conflicts with others and increases the potential for violent behavior among the drinkers and others (Wieczorek et al., 1990; Mann et al., 2006; Wahlsten et al., 2007). Expressive murders are most often preceded by arguments and altercations and the level of intoxication increases the viciousness of the attack (Karlsson, 1998). Block and Block (1992) defined expressive murders as a result of the expression, emotions, and psychological states. Emotional states such as anger, frustration, and hostility are said to lead an individual to perform expressive murders. In this context, alcohol is said to be the credible factor leading to emotional loss and instability and eventually leading to expressive-based murders. A national study of 16,698 inmates found that alcohol had a stronger role in violent offending such as homicide, physical assaults, and sexual assaults compared to offenses such as burglary and robbery. In this study, the majority of the respondents claimed to have been under the influence/intoxication of substance(s) such as alcohol during the commission of murder (Felson and Staff, 2010).

In 2011, 73 and 57% of the homicides recorded in the United States and Russia were alcohol related (Landberg and Norström, 2011), whereas, in countries including Finland, Netherlands, and Sweden, alcohol consumption led to lethal violent crimes reported from 2003 to 2006. In Finland alone, 491 persons were killed within 4 years period and ~82% of the perpetrators were intoxicated with alcohol, where 39% of them were alcoholics and 45% of the reported murders were committed with knives (Liem et al., 2013). In Singapore, out of 253 homicide offenders, 141 individuals (56%) were suffering from AUD and 121 offenders (48%) drank alcohol within 24 h preceding their criminal offense (Yeo et al., 2019). In the Brazilian city of Diadem, limiting the hours of alcoholic sales in bars to 11 p.m. significantly declined the crime rate to 9 homicides per month (Duailibi et al., 2007). Chervyakov et al. (2002) reported that 4 in every 5 Russians convicted of murder were intoxicated with alcohol during the murderous act. In a British prison sample, over a third of male homicide offenders had consumed alcohol and were considered drunk at the time of the offense and 14.0% had been using drugs (Dobash and Dobash, 2011).

Even though many findings from various countries strongly concluded that alcohol is a risk factor for murderous acts, however, most of these studies correlated level of alcohol consumption rather than the pattern of hazardous intake or types of beverages consumed, which is more likely to cause severe disinhibition, hence more damages. In line with this, using a sample of 85 countries, Weiss et al. (2018) reported no association between alcohol consumption level and homicide rates; however, they found a positive association between hazardous drinking pattern and homicide rates. Contrary to this, a cross-sectional analysis of data from 83 countries that controlled for several possible covariates reported that countries with riskier drinking patterns did not have higher homicide rates compared to countries with less risky drinking patterns. However, the same investigators also reported that the association between homicide rates and alcohol was beverage specific, with beer and spirit consumption were positively correlated with homicide rates and wine negatively correlated with the rate of homicides (Hockin et al., 2018) (Table 2).

**Table 2:** Alcohol and homicide rates.

**Study**	**Objective**	**Method**	**Sample size**	**Findings**	**Country (where the data is from)**
Gonçalves et al. (2020)	Examining the link between alcohol use and violent deaths in São Paulo, Brazil, in 2015, and its association with gender, age, cause of death, and victims' blood alcohol content (BAC).	Cross-sectional retrospective study	2,882 victims of violent death; Male > Female	Homicide was the most common cause of death in the study (36.57 percent, 1,054 cases). Nevertheless, the number of victims of road accidents was higher along with greater proportion of BAC (32.01 percent).	Brazil
Eriksson et al. (2020)	To identify how much alcohol and drugs homicide offenders used in the year preceding up to the homicide, examine the features of murder perpetrators at various degrees of problematic drug use, and investigate the features of murder incidents at different degrees of problematic drug use.	Data was acquired through face-to-face interviews in custodial and community correctional settings across Australia for this observational study.	302 individuals convicted of murder or manslaughter. (262:40: Men:Women)	Around the year leading up to the crime, many prisoners acquitted of murder or manslaughter record significant concentrations of alcohol and/or other substance abuse problems.	Australia
Bye (2008)	To see how the link between alcohol intake and homicide rates varies between nations with varied drinking habits, as well as for homicide rates by gender.	Annual alcohol consumption and homicide incidences for six eastern European nations were studied using time series analysis (Russia, Bulgaria, Poland, Hungary, Belarus and former Czechoslovakia.	–	In Eastern Europe, alcohol intake has an influence on homicide rates, which vary depending on drinking habits.	Europe
Drake (2010)	To investigate the toxicology of homicide perpetrators and victims, as well as the prevalence of homicide as a cause of death among substance users and the potential significance of treatments in lowering homicide risk.	Review of homicide toxicology and homicide as a cause of mortality among users of psychoactive substances.	–	Psychoactive drugs are significantly related to murder. The evidence indicates that this is true for both perpetrators and victims.	Australia
Forsman et al. (2018)	The goal was to use toxicological data from homicide victims and offenders, as well as controls who died in vehicle-related accidents, to assess the risks of homicide offending and victimization given by the presence of ethanol in blood.	Forensic toxicological results were gathered from official registries and databases across the country.	Homicide victims: 200 Homicide offenders: 105 Individuals killed in vehicle related accidents: 1,629	Homicide offense and victimization are attributed to alcohol intake.	Sweden
Landberg and Norström (2011)	To compare and contrast the aggregate association between alcohol and homicide in Russia and the United States.	For the age ranges 15–64 years, 15–34 years, and 35–64 years, researchers looked at overall and sex-specific homicide rates. For Russia, the study period was 1959–1998 and for the United States, 1950–2002. Alcohol consumption in the United States was measured by alcohol sales, while in Russia, estimated unrecorded consumption was also included. Autoregressive integrated moving average (ARIMA) was used to evaluate the data.	–	A 1-L rise in intake was correlated to a 10% increase in homicides in both Russia and the United States. However, the absolute impact was found to be even higher in Russia than America, due to disparities in homicide rates.	Russia, America.
Kuhns et al. (2014)	To calculate the percentage of homicide suspects who tested positive for alcohol and/or were inebriated at the time of the crime	Meta-analysis of 23 independent studies	28,265 homicide offenders from nine different countries	Homicide rates are strongly linked to alcohol intake levels in total.	America
Lira et al. (2019)	To describe primary and corollary IPV homicide victims in relation to BACs, and to see if a 10% increase in the restrictiveness of the alcohol policy environment was linked to a lower risk of alcohol involvement among IPV homicide victims.	This was a repeated cross-sectional study that examined the association between alcohol policies and alcohol participation in IPV homicide victims in the United States.	26,974 homicide victims with BAC testing	Alcohol consumption was common among Intimate Partner Violence - Homicide victims. Moreover, more stringent alcohol laws were linked to a lower risk of alcohol involvement.	America
Branas et al. (2016)	Exploratory study	For the 40 years from 1975 to 2014, a systematic literature review with meta-analysis of the link between alcohol and weapons was conducted.	–	Alcohol is used or misused by a significant proportion of firearm injury victims and owners. Alcohol consumption firearm violence are so intricately connected which thus can make alcohol a modifiable risk factor in the prevention of firearm violence.	America
Naimi et al. (2017)	To investigate the relationships between the alcohol policy environment and alcohol use among homicide victims in the United States, both generally and by sociodemographic groupings	The Alcohol Policy Scale (APS) ratings were calculated using 29 alcohol regulations by state and year. The National Violent Death Reporting System provided information on homicide victims in 17 states from 2003 to 2012. Scores from the APS were used.	–	Lower risks of becoming a victim of an alcohol-related homicide overall and within the groups at high risk of homicide are strongly correlated to more stringent alcohol regulation conditions. An effective homicide-prevention approach is to strengthen alcohol policies.	America
Rossow (2001)	To determine an empirical basis for cross-national and cross-cultural comparisons of few aspects of the relationship between alcohol consumption and homicide	For each country, time series analyses were done using differenced series of annual aggregate-level data on alcohol sales and homicide rates from 1950 to 1995 (ARIMA-models).	–	Alcohol sales have an impact on homicide rates, especially in northern European countries where drinking culture is marked by heavy drinking incidents to a greater degree.	Europe
Rossow (2004)	To determine a scientific evidence for cross-province comparisons of two components of the alcohol-violence relationship: the relative strength of the relationship and any gender disparities in the relationship between consumption and victim rates	Data analysis using ARIMA model	–	Homicide rates are significantly affected by alcohol sales, especially in some provinces and among men.	Canada
Swart et al. (2015)	To describe the blood alcohol concentration (BAC) of juvenile homicide victims in Johannesburg, South Africa, and to identify the victim and event features linked to a high BAC at the time of death.	The National Injury Mortality Surveillance System's mortality data was analyzed using logistic regression (NIMSS).	323 homicide victims (15–19 years old) with the presence of alcohol	In Johannesburg, South Africa, significant rate of adolescent killings is strongly linked to heavy alcohol intake. It is more common among male and older adolescent victims, as well as those killed with sharp objects during weekends and evenings.	South Africa
Trangenstein et al. (2020)	To examine the link between alcohol marketing seen outside off-premise alcohol businesses and violent crime.	For multiple testing, the authors utilized mixed models with a Simes-Benjamini-Hochberg adjustment.		The association between the outlets and violent crime was positively correlated. The prevalence of alcohol ads outside off-premises outlets was attributed to an increase in violent crime.	America
Yeo et al. (2019)	To find out how common alcohol use disorders are among those charged with homicide in Singapore.	To account for known confounding variables and investigate the link between alcohol usage and homicide, regression models were utilized.	253 homicide offenders; 149 individuals with psychiatric diagnosis	In Singapore, alcohol use disorders are perhaps the most prevalent condition found in murder suspects. Homicide perpetrators with Severe Mental Illness are less likely to consume alcohol.	Singapore

## Alcohol and Aggression: A Neuroscience Perspective

Alcohol accentuates or promotes the mental state of the drinkers at the time of consumption, fueling negative emotions such as aggressive behavior or positive emotional outcomes such as gregariousness and warmth. Aggression is classified as impulsive, premeditated, and medically driven (Gollan et al., 2005). Even cognitively intact alcohol-dependent individuals showed higher psychopathological symptoms with trait impulsivity (Kovács et al., 2020) and other psychiatric comorbidities such as antisocial and borderline personalities (Helle et al., 2019) triggering medically driven aggression. Unlike impulse-driven aggression, which is reflective of an agitated state of mind, premeditated aggression is a planned aggressive act (Martin et al., 2019).

The aggressive acts at some points could be goal oriented, whereas in some instances could be impulse driven. Impulsivity is defined as fast actions taken without adequate or with little thought and conscious judgment of the consequences (Bakhshani, 2014). Assessment of various brain regions of 1,200 men and women of 18–35 years old along with their tendency to make rapid decisions seek for novel and intense experiences and risk-taking traits revealed a significant decrease in the cortical thickness of brain regions related to self-control and decision-making processes, particularly anterior cingulate and middle frontal gyrus (Holmes et al., 2016). Alcohol itself directly interrupts the executive cognitive functions by disrupting the functions of the prefrontal cortex (PFC), which has been associated with disinhibition and aggression (Heinrichs, 1989). The PFC, which regulates aggressive and social behavior (Davidson et al., 2000; Seo et al., 2008), was shown to be reduced in its volume in individuals with antisocial personality disorder (Raine et al., 2000). In addition, neuroimaging of individuals with IED revealed lower white matter integrity in long-range connections between the frontal and temporoparietal regions (Lee et al., 2016), reduced gray matter volume in the frontolimbic structures (Coccaro et al., 2016), and gray matter deficit and dysfunction of the left insula (Seok and Cheong, 2020). The orbitomedial region within the PFC regulates anger and impulsive aggression (Lapierre et al., 1995; Davidson et al., 2000) and assigns appropriate emotion to the consequences of actions (Bechara et al., 2000). During aggressive behaviors, reduced activity was reported within the orbitofrontal PFC (Goyer et al., 1994; Pietrini et al., 2000), where the impaired PFC is unable to inhibit the subcortical structures such as the amygdala, hippocampus, and nucleus accumbens from activating emotional output (Raine et al., 1998; Davidson et al., 2000). In line with this, an increase in amygdala limbic connectivity and a significant decrease in amygdala-medial PFC connectivity were reported among violent offenders (Siep et al., 2019). Hyperactivation of the amygdala is also reported in individuals with IED in response to angry faces compared to controls (McCloskey et al., 2016). Moreover, alcohol-dependent patients with a history of aggressive behavior also recorded elevated glutamate/creatine ratio in the bilateral amygdala (Liu et al., 2020) corroborating various other behavioral changes associated with glutamatergic hyperexcitability state in the amygdala reported in past studies (Kumar et al., 2013, 2016, 2018; Pakri Mohamed et al., 2018; Kamal et al., 2020).

## Serotonin in AUD and Aggression

Aggression is a complex behavior involving interactions between the gene, environment, personality, and physiology (Armstrong et al., 2017; Zhang et al., 2017; Kanen et al., 2021). Dysregulation of serotonin is associated with many psychiatric disorders (Rappek et al., 2018; Conio et al., 2020; Fanning et al., 2020) due to the widespread distribution of serotonergic fibers originating from midbrain raphe nuclei to various other regions (Sharp and Barnes, 2020). Based on a systematic review, the association between serotonin and aggression is rather mixed, where reduced 5-hydroxytryptamine (5-HT) concentration in central nervous system (CNS) was associated with reactive aggression (impulsivity; response to provocation), whereas increased 5-HT (small number of findings) may be related to callous-unemotional traits, which is another possible pathway to aggressive behavior (Runions et al., 2019). In line with this, SLC6A4^*^HTTLPR or 5-HTTLPR (serotonin transporter) was associated with aggression within the population of Pakistan (Qadeer et al., 2021), China (Zhang et al., 2017), and the United States of America (Armstrong et al., 2017), whereas, in a study conducted among Russian inmates, such correlation was not found (Toshchakova et al., 2017). Furthermore, other genes of serotonin such as 5-hydroxytryptamine receptor 2A (5HTR2A), 5-hydroxytryptamine receptor 2B (5HTR2B), and 5-hydroxytryptamine receptor 2C (5HTR2C) also showed no association with aggressive behavior (Toshchakova et al., 2017; Qadeer et al., 2021), suggesting a stronger link between brain serotonin level and aggression rather than the receptors, which was also proven by studies using selective serotonin reuptake inhibitors (SSRIs) (Nord et al., 2013; Lagerberg et al., 2020). Likewise, a lower cerebrospinal level of 5-hydroxyindoleacetic acid (5-HIAA), the main metabolite of serotonin, was reported in the impulsive offenders than the premeditated murderers (Linnoila et al., 1983). Regions such as the cingulate cortex, ventromedial, and the orbitofrontal PFC were shown to have reduced serotonergic activity during impulsive aggression (Siever et al., 1999). Similar findings also reproduced in non-human animal models (Harrison et al., 1997; Kästner et al., 2019; Gorlova et al., 2020).

Some researchers have reported high serotonin transporter (SERT) bindings in the brains of deceased alcoholics (Underwood et al., 2018), whereas others have reported low binding (Mantere et al., 2002) and some reported no differences (Brown et al., 2007; Martinez et al., 2009). Similarly, mixed findings were also reported for 5-HT1A and 5-HT2A receptor bindings (Underwood et al., 2008, 2018; Storvik et al., 2009). Chronic alcohol intake increases the metabolites of serotonin in the raphe nuclei area, however reduces 5-HT2A protein levels in the mice cortex, indicating reduced serotonergic activity (Popova et al., 2020). Acute alcohol intake reduces tryptophan availability to the brain (non-aggressive), which leads to a decrease in serotonin synthesis and turnover, about 25% of the concentration of tryptophan following an oral intake of alcohol (Badawy et al., 1995). Hence, it is probable that in the aggressive brain, the drop in brain serotonin synthesis might even be greater (40–60%) during moderate intake of alcohol (Badawy, 2003). However, the inconsistent findings of serotonin markers in brain imaging studies of alcoholics suggest that comorbidity of AUD with other psychiatric disorders may complicate the serotonin hypothesis in real life. In addition, even individual differences in personality traits determine the types of emotion affected by the depletion of serotonin (Kanen et al., 2021).

## Dopamine in AUD And Aggression

Serotonin and dopamine levels are significant predictors of aggression and suicide risk (Prepelita et al., 2019). A systematic review of pre-clinical findings suggests that adolescence chronic stress may lead to a hyperdopaminergic state of the PFC, which eventually blunts the adulthood prefrontal dopaminergic neurotransmission, increasing the vulnerability to maladaptive aggression in adulthood (Tielbeek et al., 2018). In relation to this, polymorphisms of catecholamine-converting enzymes such as monoamine oxidase and catechol-o-methyltransferase along with traumatic childhood significantly increase appetitive and Facilitative Aggression Scale (Fritz et al., 2021). Furthermore, a study conducted on convicted Pakistani murderers revealed a high prevalence of the 9R allele of DAT-1VNTR, which influences the intrasynaptic dopamine levels (Qadeer et al., 2017). Pharmacological modulation of dopamine D2 receptor *via* its antagonist, sulpiride, impaired the ability to discern angry facial expressions in humans (Lawrence et al., 2002). However, some researchers have reported the opposite, where polymorphism in DRD2 genotypes causes reduced dopamine functioning that is directly associated with increased aggression (Zai et al., 2012) which may occur through sensation seeking (Chester et al., 2016). Nevertheless, it was hypothesized that impaired serotonin neuromodulatory effects may lead to dopamine hyperactivity in subcortical structures and aggressive behaviors (Seo et al., 2008). Studies investigating the interaction between genetic polymorphism of dopamine system (dopamine receptors; DRD2, DRD4, transporter; DAT1), and environmental factors (financial stressor and adolescent social experiences) on intimate partner violence revealed a strong influence of negative environmental changes on increased odds of violence perpetration regardless of the alleles (Schwab-Reese et al., 2020).

In addition to aggression, alcohol alone modulates dopaminergic neurotransmission, where even the cues of alcohol could increase the dopamine release in the nucleus accumbens (Melendez et al., 2002). Dysregulation of dopaminergic neurotransmission in AUD has been demonstrated in several brain imaging studies (Leurquin-Sterk et al., 2018; Chukwueke et al., 2021). Factors such as personality traits and comorbidities with other psychiatric disorders along with environmental stressors influence how one could engage in violent behaviors. Hence, even though alcohol might be the precursor to violence for some, it certainly takes more than the beverage to increase the likelihood of someone shooting from the hip.

## Alcohol, Aggression, and Violence: A Conundrum

Individual reports from multiple countries have associated alcohol with violent crimes and domestic abuse. Consumption of alcoholic beverages with higher alcohol content at a dose of 0.75 g/kg and higher was correlated with increased aggression (Hockin et al., 2018; Kuypers et al., 2020), whereas a comprehensive review found no association between homicide rates and alcohol consumption level (Weiss et al., 2018). Even countries with a riskier drinking pattern did not show a higher crime rate compared to countries with less risky drinking patterns (Hockin et al., 2018). This led us to the question, does alcohol alone is sufficient to trigger violent or aggressive behavior? Based on the pieces of literature gathered in this article and past findings, it is evident that several individual and environmental factors determine the likelihood of an intoxicated person engaging in an aggressive or violent act. Emotional dysregulation and impulsivity in combination with pre-existing psychiatric comorbidities such as personality disorders, intermittent explosive disorder along with genetic pre-disposition and environmental stressors, such as the most commonly associated childhood adversity, are one of the triggers of intoxicated aggression. Genetic polymorphism findings indicate that environmental stressors play a more significant role in perpetration violence compared to high-risk genotypes (Schwab-Reese et al., 2020). However, some have reported that epigenetic mechanisms mediate the interaction between genetic and environmental factors by altering genes of many systems including the nervous, immune, and neuroendocrine (Chistiakov and Chekhonin, 2017).

**Figure 1:**
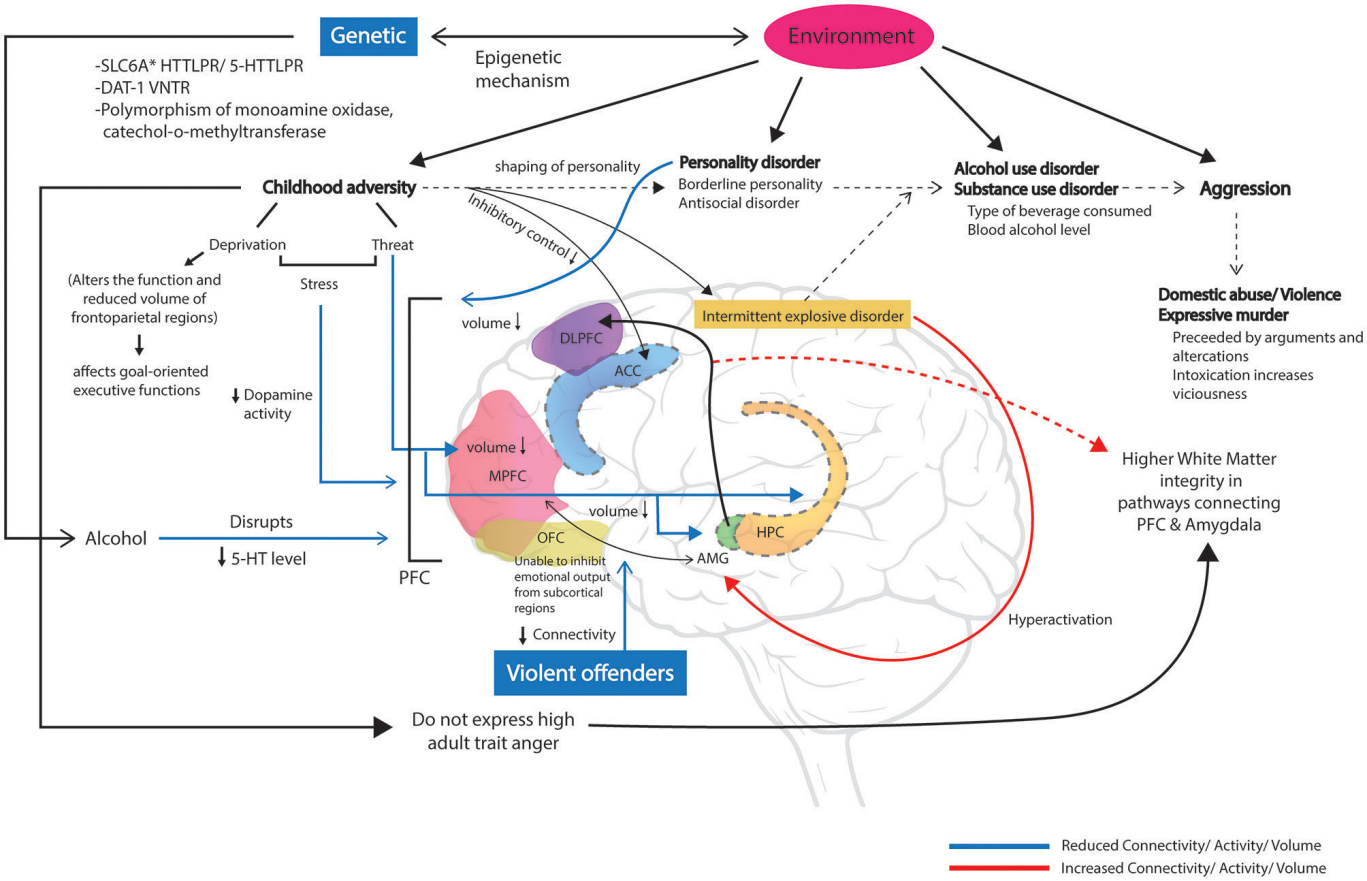
Childhood adversity affects the shaping of personality, which eventually leads to development of personality disorder, alcohol use disorder, substance use disorder, intermittent explosive disorder (IED), and aggressive behaviors such as domestic abuse or expressive murders in adulthood. Factors such as genetics and environment also interact with alcohol intake and causing neuroplasticity in brain regions associated with emotional and cognitive regulation. Childhood stress such as deprivation alters the function and reduces the volume of frontoparietal regions that associated with goal-oriented and executive functions. Childhood adversity including threat reduces the volume of the medial prefrontal cortex (MPFC), amygdala (AMG), and hippocampus (HPC). Chronic early life stress also blunts the dopaminergic activity in the PFC. Alcohol disrupts the serotonergic activity in the PFC. Altered functions of the orbitofrontal cortex (OFC) unable to inhibit the increased emotional output from subcortical structures such as the hyperactivation of AMG in IED. Reduced connectivity between MPFC and AMG was reported in violent offenders. Adults having experienced childhood adversity and do not express high adult trait anger were due to higher white matter integrity in pathways connecting the PFC and AMG.

Stress during early life, also known as childhood adversity or childhood maltreatment, is associated with the development of personality disorders (Lemgruber and Juruena, 2013; Porter et al., 2020), affective disorders (Hoppen and Chalder, 2018), and alcohol use disorder (Evans et al., 2017). Among these, physical, emotional abuse, and maternal rejection are associated with the shaping of personality (Schouw et al., 2020) and maladaptive schemes in adulthood (Pilkington et al., 2021). For an instance, physical abuse and neglect lead to antisocial traits (Schorr et al., 2020). Factors such as family dysfunction, as violence in the family, show a strong correlation with adulthood aggression (Khodabandeh et al., 2018; Labella and Masten, 2018) through emotion-related impulsivity and behavioral response inhibition (Madole et al., 2020). In line with this, it has been reported that a high level of childhood adversity increases one's likelihood to substance use through reduced functioning of the anterior cingulate cortex in inhibitory control, indicating a higher impulsive response (Fava et al., 2019). The very nature of adversity (threat vs. deprivation) has a distinctive effect on emotional circuits. For an instance, childhood threat was reported to reduce the volume of the medial PFC, amygdala, and hippocampus along with increased activation of the amygdala in response to a threat, whereas childhood deprivation alters the function and volume of the frontoparietal regions, which are associated with goal oriented and executive functions (McLaughlin et al., 2019). In addition to the type of adversities, individual differences in threat and executive control-related brain regions also determine how one with childhood adversity would express adult trait anger. Individuals with the low amygdala and high dorsolateral PFC activity do not express high trait anger, despite having experienced stress in early life (Kim et al., 2018). Suppression of adult trait anger was owing to the higher microstructural integrity of white matter pathways, including the uncinate fasciculus, which anatomically links the PFC and amygdala in the regulation of negative emotion (Kim et al., 2019). However, the findings by Kim et al. (2019) were based on subjects free of borderline and personality disorders, which are the most commonly associated psychiatric comorbidities with intoxicated aggression and also known to have reduced white matter integrity in regions associated with risky behavior and impulsivity (Jiang et al., 2017; Ninomiya et al., 2018). Hence, more longitudinal studies are needed in the future to understand the effects of early life stress on the development of aggression-related psychiatric comorbidities from neurological perspectives. Furthermore, the role of white matter integrity in one's expression of anger despite the chronic stress in early life should be further explored to understand the cause behind such discrepancy and the consistent neurological changes noticed in conjunction with high-risk behaviors could be investigated as potential biomarkers to predict one's risk factor along with social experiences (Figure 1).

## Gender Differences in Binge Drinking, Alcohol-Induced Aggression, and Violence

It was initially reported that women are less likely to engage in binge drinking patterns than men (Bobrova et al., 2010). However, in the recent years, data from the United States indicate that the binge-drinking rate in adult women (age 21–49 years) has been rising (Hasin et al., 2019; Sarah and Keyes, 2020). Evidence suggests that there is a little convergence in the pattern of binge drinking in men and women. It was found that the prevalence was higher for females than males from 2000 to 2010 for any binge drinking in the preceding month. On the contrary, the reason for the convergence of frequency in the male and female binge drinking habits is estimated to occur due to the large decline in the binge drinking frequency within men than the women. Furthermore, evidence also shows that the convergence of men and women has usually been stronger in the age group of young adults in comparison to any other age group (Wilsnack et al., 2018). Data from 2006 to 2018 indicate that both the men and women increasingly binge drink; in women, the largest increase was found in the age group 30–44 years without children (Sarah and Keyes, 2019).

Several studies have investigated the risk factors pertaining to intimate partner violence (IPV)/domestic violence (DV) and found that gender-specific differences exist in DV. The likelihood of females being victimized is greater than the male victimization, whereas evidence for the males being the perpetrators is higher than that of the females. The risk factors that are found to be common in both the men and women reporting perpetration involved being exposed to parental violence and physical abuse during childhood and alcohol abuse. Risk factors that are thought to be associated with male perpetration include unemployment, lower income, cohabitation, mood disorders, and no or lower level of education (Gass et al., 2011; Lee et al., 2014). Lack of education is thought to play a role in both the perpetration and victimization of women (Capaldi et al., 2012). Moreover, factors such as pregnancy, young age, higher income than the partner, and previous relationships increase the risk of victimization for women (Capaldi et al., 2012).

Prior reports have established alcohol-induced aggression among males (Lipsey et al., 1997), which appears to vary across the ethnic groups and geographical regions (Caetano et al., 2001). Systematic comparison between males and females in relation to alcohol-induced aggression revealed greater effects of alcohol on males than females (Ito et al., 1996; Bushman, 2002); however, the analysis was limited by insufficient power to detect significant effects due to limited female data. In agreement with this, a separate study reported a small-to-moderate effect size for the association between alcohol use and male-to-female partner violence, whereas a small effect size for the association between alcohol intake and female-to-male partner violence (Foran and O'Leary, 2008). More recently, a significant, small effect size was reported for the association between alcohol intake and aggression in female subjects who consumed alcohol compared to those who did not drink, in response to a subsequent aggression paradigm (Crane et al., 2017).

Males are more likely to express aggression in a physical and/or direct form, whereas females are more likely to express it in an indirect form. It has also been reported that both the males and females are equally aggressive when verbal aggression is at play (Archer, 2004; Björkqvist, 2017). In an experiment conducted by Giancola and Zeichner (1995), 128 participants (64 males and 64 females) performed a task where they gave an electric shock to the fictional opponents, which included both the genders. The participants were assigned to either alcohol, a placebo, or a sober group. The researchers found that the intensity and duration of shock were higher in the men from the alcohol group, while only shock duration was increased in women. They also noted that men were highly aggressive toward the same gender, while women were aggressive regardless of gender. This indicated that alcohol-induced aggression affects both the genders in different ways, suggesting that men are likely to respond in a direct and indirect manner, whereas women exhibit aggression in an indirect manner. A slightly different finding to the previous study was seen in an investigation conducted by Hoaken and Pihl (2000). The researchers assigned the participants (54 males and 60 females) to compete in a competitive aggression paradigm in an intoxicated or sober state. The result was that the intoxicated men were more aggressive than the sober men; however, in the circumstances where the women were highly provoked, both the intoxicated and sober women displayed higher levels of aggression, which could resemble the men. This suggested that both the women and men can be equally aggressive and alcohol does not seem to play a prominent role in the gender biases in aggression.

Several brain imaging studies have examined the neurological changes in men and women during aggression either by including an equal number (almost) of male and female subjects or a single gender (against a control group) (Chester and DeWall, 2016; Emmerling et al., 2016; Denson et al., 2018). To date, very few studies have tested the gender difference hypothesis using both the male and female subjects. Generally, men have recorded higher activation of the amygdala (McRae et al., 2008) and the PFCs (Rahko et al., 2010) during emotional reactions. Investigation of sex differences in neural correlates of aggression using 22 male and 20 female subjects revealed differential brain activation patterns between both the genders in response to provocation. Aggressive men recorded higher activation of the left amygdala than aggressive women and a positive correlation with orbitofrontal cortex (OFC), rectal gyrus, and ACC activity, which was negatively correlated in women. The findings indicate that aggressive men are more inclined to automatic emotion regulation (attributed to OFC and rectal gyrus) in response to provocation compared to aggressive women (Repple et al., 2018). In a separate study involving 24 men and 11 women, alcohol alone had no effect on the amygdala and ventral striatum; however, their activities were positively correlated with aggression in response to provocation. Alcohol decreased their bold responses in the right PFC, thalamus, hippocampus, caudate, and putamen. Neither gender had any significant impact on the results (Gan et al., 2015). Contrary to this, a single administration of 0.5 per thousand alcohol was shown to reduce frontal interhemispheric connectivity in female participants, but not in male participants (Hoppenbrouwers et al., 2010). Intergender neurological and behavioral responses to alcohol are also influenced by ethanol metabolism (Arthur et al., 1984) and influences of hormones such as testosterone, cortisol, estradiol, progesterone, and oxytocin (Denson et al., 2018).

## Conclusion

Alcohol intoxication-induced aggression is an interplay between gender, genetic, psychiatric comorbidities, blood alcohol level, and environmental factors. Risk factors associated with intoxicated aggression or aggression should be packaged into a scientific explanation to educate the public. Alcohol is a weak drug, which needs to be consumed in large amounts in order to cause intoxication. Hence, high-risk individuals should practice moderate drinking. Parental roles in shaping the personalities of children should be incorporated into the marriage course as one of the preventive measures. Future studies and policymakers should include more behavioral interventions in the high-risk adolescent groups.

## Author Contributions

KS and JK contributed to the conceptual framework, design, and drafted the manuscript. MR, IN, RM, and MS searched references and critically revised the manuscript. HK prepared the figure and legend. All the authors critically reviewed content and approved the final version for publication of manuscript.

## Funding

This study was funded by the Ministry of Higher Education Malaysia, FRGS/1/2020/SKK0/UKM/02/3.

## Conflict of Interest

The authors declare that the research was conducted in the absence of any commercial or financial relationships that could be construed as a potential conflict of interest.

## Publisher's Note

All claims expressed in this article are solely those of the authors and do not necessarily represent those of their affiliated organizations, or those of the publisher, the editors and the reviewers. Any product that may be evaluated in this article, or claim that may be made by its manufacturer, is not guaranteed or endorsed by the publisher.
